# Editorial: The use of deep learning in mapping and diagnosis of cancers

**DOI:** 10.3389/fonc.2022.1077341

**Published:** 2022-12-13

**Authors:** Abhishek Mahajan, Nivedita Chakrabarty

**Affiliations:** ^1^ Department of Radiology, The Clatterbridge Cancer Liverpool, Liverpool, United Kingdom; ^2^ Department of Radiodiagnosis, Tata Memorial Hospital, Homi Bhabha National Institute (HBNI), Mumbai, India

**Keywords:** deep learning - artificial neural network, artificial intelligence-AI, oncology, cancer imaging and image-directed interventions, systematic review and meta-analysis, original research article, machine learning and AI, oncoimaging

Deep Learning (DL) is a subset and an augmented version of Machine Learning (ML), which in turn is a subgroup of Artificial Intelligence (AI), that uses layers of neural networks, similar to human brain, for performing complex tasks quickly and accurately. AI can recognize patterns in a large volume of data and extract characteristics imperceptible to the human eye (1). Convolutional Neural Network (CNN) is the most commonly used network of DL, which contains multiple layers, with weighted connections between neurons that are trained iteratively to improve performance. DL can be supervised or unsupervised, but most of the practical uses of DL in cancer has been with supervised learning where labelled images are used for data training (2). Despite the growing number of uses of DL in cancer mapping and diagnosis, there are uncharted territories in DL which remain to be explored to utilize it to its full capacity. Also, in spite of the revolution in cancer research that DL has ushered in, there are a lot of challenges to overcome, before DL can be widely used and accepted in every corner of the world.

## Role of DL in oncology

There has been an unprecedented surge in DL based research in oncology due to the availability of big data, powerful hardware and robust algorithms. Screening and diagnosis of cancer, prediction of treatment response, and survival outcome and recurrence prediction, are the various roles of ML and DL in cancer management. AI algorithms integrated with clinical decision support (CDS) tools can automatically mine electronic health record (EHR) and identify cohort that would benefit maximum from cancer screening programmes (3). For successful implementation of AI in cancer diagnosis, it is imperative for the radiologists and pathologists to collaborate with the key stakeholders, industrial partners and scientists (4). With ever increasing cancer burden worldwide, and availability of molecular targeted therapies, DL has served as an elixir, by its ability to screen, detect and diagnose tumours rapidly, and predict biomarkers non-invasively on imaging (5). Studies have shown that DL can be used to stage and grade tumours quickly and provide non-invasive histopathological diagnosis in cases where obtaining an invasive sample is risky. Patients, clinicians, radiologists and the pathologists, all have the potential to be benefitted by this DL technology as the utility of DL is no longer limited to tumour diagnosis, but to the cancer care as a whole. Prediction of overall survival, progression free survival, and disease free survival, assessment of response to treatment and outcome prediction are few of the many ways DL can benefit patients afflicted with cancer, the mere thought of which was previously unfathomable (5). Treatment planning and patient management can be hastened through the wider applications of DL based image interpretation, for example, non-responders to treatment detected on DL based baseline image interpretation, can be spared of further invasive treatment, and a change in management strategy may be considered for them.

## Major applied uses of DL technology

### Image classification and regression

DL can be used for classifying a lesion into benign or malignant, for treatment response evaluation and survival prediction. If DL models can be trained using a large dataset from a source domain, then it can be used in a target domain with a small sample size (2).

### Object detection

DL can be used in tumour localization.

### Semantic segmentation

DL can mark specific areas of concern on an image and assist the radiologists in decision making (2).

### Image registration

Images acquired at different times can be accurately linked using DL, thus, enabling the radiologists to compare the images (2).

### Federated learning

Robust deployable model can be built notwithstanding geographic boundaries, if multiple organizations/institutions/hospitals jointly train a model on a large data after de-identification of patient information (6).

## Systematic review and meta-analysis data

A systematic review and meta-analysis from 1^st^ January 2012 to 6^th^ June 2019, comparing the diagnostic accuracy of health-care professionals with deep learning algorithms using imaging, found 10 studies on breast cancer, 9 studies on skin cancer, 7 studies on lung cancer, 5 studies on gastroenterological or hepatological cancers, 4 studies on thyroid cancer, 2 studies on oral cancer, and 1 study on nasopharyngeal cancer (7). Another systematic review on AI techniques in cancer diagnosis and prediction from articles published from 2009 to April 2021, revealed 10 articles pertaining to brain tumours, 13 articles related to breast cancer, 8 articles each related to cervical, liver, lung, and skin cancers, 6 articles related to colorectal cancer, 5 articles each related to renal and thyroid cancers, 2 articles each related to oral and prostate cancers, 7 articles related to stomach cancer, and 1 article each related to neuroendocrine tumours and lymph node metastasis (8). Few studies involving AI in cancer diagnosis and management include:

- Histology prediction and screening of breast cancer on mammography (9, 10).
- Brain tumour segmentation (11–14).
- Lung nodule segmentation on computed tomography (CT) (15–17).
- Liver tumour segmentation on CT (17, 18).
- Prostate gland tumour detection on magnetic resonance imaging (MRI) (19, 20).
- Brain tumour survival prediction (21–23).
- F. Glioblastoma recurrence prediction (24).

## Challenges and limitations of DL

- Requirement of a large data: DL models need a large data (in thousands) to be trained and availability of such a huge data may not be possible in every institution.
- Precise data annotation: Tumour region needs to be annotated or labelled accurately without contamination from surrounding non-tumour regions. This may not always be possible as many a times, tumours are infiltrative in nature and not discrete, and may be located within a region containing some other pathology, for example, infiltrative lung tumour located within a collapsed lung, in which case precise margin delineation may not be possible.
- There is need for equal representation of data on training and test sets failing which data gets skewed and bias is introduced (2).
- Heterogeneity of data: Difference in training set of images and deployable image sets may affect the performance of a model, for example if the CT scanner used while acquiring images for training is different from the one on which the model is validated, then performance may be reduced.
- Patient privacy concerns: Despite the available methods for deidentification of patient information, the problems of patient privacy still loom large (2).
- Problem of hidden layers: DL uses multiple layers of neural network to analyse data, which remain hidden, and the exact reasoning of outcome is not decipherable, which makes it difficult to be relied upon and convincingly used.
- Infrastructure: Use of DL requires a robust infrastructure which may not be available everywhere.
- Lack of trained personnel and expertise and lack of awareness about collaboration for implementation of AI projects (25).

## Imaging biobanks

Repositories of human tissue sample stored in an organized manner for research purpose is known as “biobank”, and collection of medical image data for long term storage and retrieval for research is known as “imaging biobank” (26, 27) Digital Imaging and Communications in Medicine (DICOM) is the universal format for Picture Archiving and Communication System (PACS) storage and data sharing across all institutions (26). The data needs to be de-identified and informed consent of the patient obtained prior to data archiving (28). Few examples of such open-source platforms include The Cancer Genome Atlas (TCGA) program, The Cancer Imaging Archive (TCIA), and European Genome–phenome Archive (EGA) (29, 30). In India, collaboration between the Department of Biotechnology (Government of India) under the guidance of the National Institution for Transforming India (NITI) Aayog, and Tata Memorial Centre has led to the creation of The Tata Memorial Center Imaging Biobank (31). World’s biggest multi-modality imaging study was commenced by the UK Biobank in 2014 to have a repository of neuro, cardiac, and abdominal MRI imaging, dual energy x-ray absorptiometry (DEXA) and carotid ultrasonography (32). Similarly, CAN-I-AID (Cancer Imaging Artificial Intelligence Database) biobank project has been initiated by Dr. Abhishek Mahajan at the Clatterbridge Cancer Centre, Liverpool, United Kingdom (UK). Such imaging biobanks for public use should be encouraged as it fulfils the requirement of large image data to promote DL based research across the globe.

## Articles in research topic

**Figure 1:**
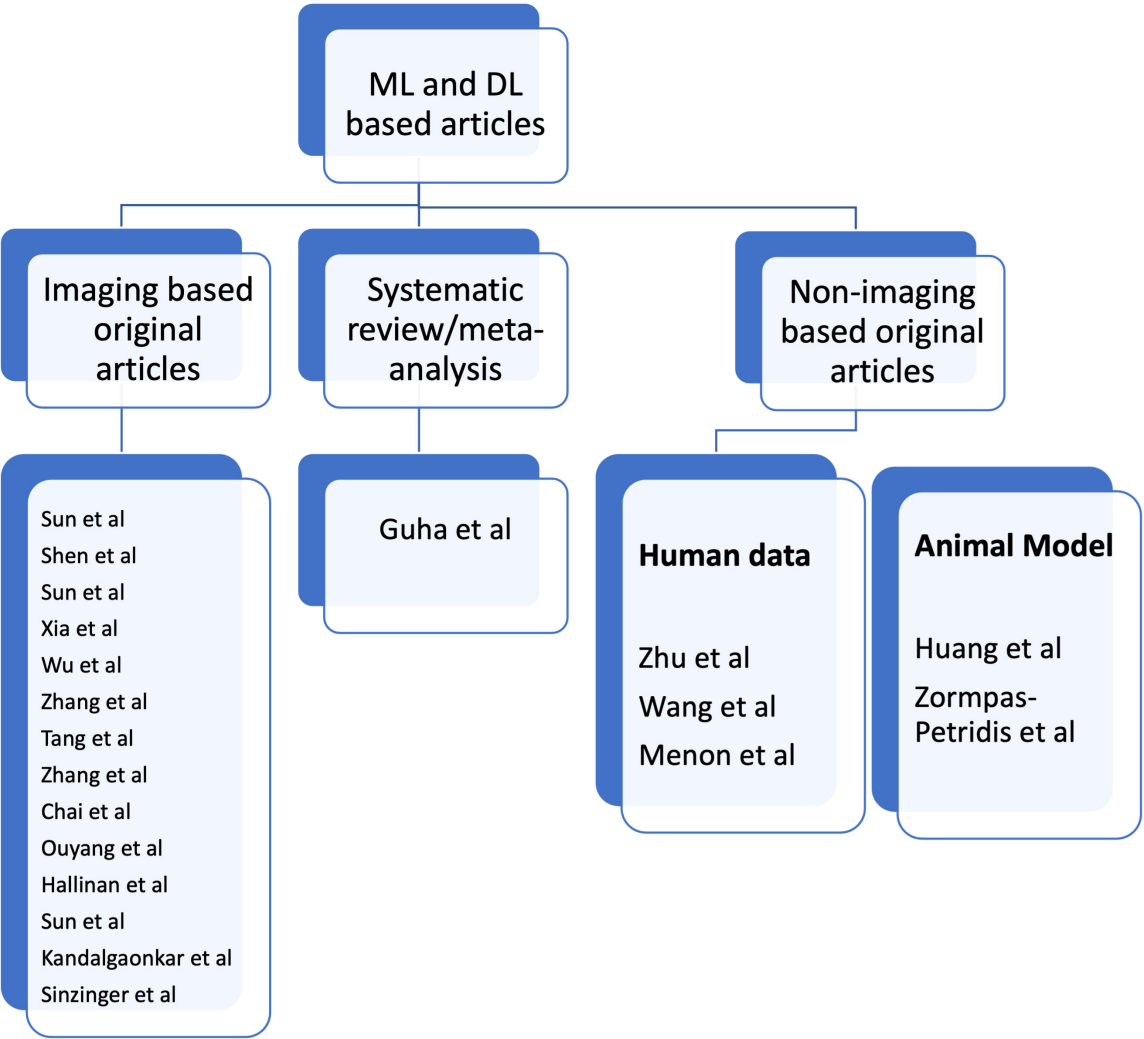
List of authors based on type of articles submitted towards research topic.

In this Research Topic, we present 20 topics, 19 of which are original articles and one is a systematic review. *There is one article on cervical cancer screening:* Sun et al. used Stacking-Integrated Machine Learning Algorithm based on demographic, behavioural, and clinical factors to accurately identify women at high risk of developing cervical cancer and suggested the use of this model to personalise cervical cancer screening programme. *Three articles on lung cancer*: Shen et al. showed that DL based CT images have the potential to accurately predict malignancy and invasiveness of pulmonary subsolid nodules on CT Images and thus aid in management decisions. Sun et al. conducted a study to establish the role of Convolutional Neural Network-Based Diagnostic Model to differentiate between benign and malignant lesions manifesting as a solid, indeterminate solitary pulmonary nodule (SPN) or mass (SPM) on computed tomography (CT). Xia et al. compared and fused DL and Radiomics features of ground-glass nodules to predict the invasiveness risk of stage-I lung adenocarcinomas in CT scan and concluded that fusion of DL and radiomics features can refine the classification performance for differentiating non-invasive adenocarcinoma (non-IA) from IA and the prediction of invasiveness risk of GGNs is similar to or better than radiologists using AI scheme. *One article on thyroid cancer:* Wu et al. combined ACR TI-RADS with DL by training three commonly used deep learning algorithms to differentiate between benign and malignant in TR4 and TR5 thyroid nodules with available pathology and concluded that irrespective of the type of TI-RADS used for the classification competition, DL algorithms outperformed radiologists. *One article on bladder cancer:* Zhang et al. proposed a DL model based on CT images to predict muscle-invasive status of bladder carcinoma pre-operatively and concluded that DL model exhibited relatively good prediction ability with capability to enhance individual treatment of bladder carcinoma. *One article on periampullary region:* Tang et al. used DL to identify periampullary regions on MRI images and achieved optimal accuracies in the segmentation of the peri-ampullary regions on both T1 and T2 MRI images concordant with manual human assessment. *One article on rectal cancer:* Zhang et al. segmented rectal cancer *via* 3D V-Net on T2WI and DWI and then compared the radiomics performance in predicting KRAS/NRAS/BRAF status between DL-based auto segmentation and manual-based segmentation. They concluded that 3D V-Net architecture could conduct reliable rectal cancer segmentation on T2WI and DWI images. *One article on jaw lesions:* Chai et al. showed that AI-based cone-beam CT can distinguish between Ameloblastoma and Odontogenic Keratocyst with better accuracy than the surgeons. *Two articles on spine:* Ouyang et al. evaluated the efficiency of DL-based automated detection of primary spine tumours on MRI using the turing test. Hallinan et al. developed a DL model for classifying metastatic epidural spinal cord compression on MRI and which had comparable agreement to a subspecialist radiologist and clinical specialists. *One article on kidney tumour:* Sun et al. conducted a study on kidney tumour segmentation based on FR2PAttU-Net model. *One article on brain tumour:* Kandalgaonkar et al. conducted a study predicting IDH subtype of Grade 4 Astrocytoma and Glioblastoma from tumour radiomic patterns extracted from Multiparametric MRI using a machine learning approach and inferred that it may be used in either escalating or de-escalating adjuvant therapy for gliomas or for using targeted agents in future. *One article on survival rate prediction in cancer patients:* Sinzinger et al. developed Spherical Convolutional Neural Networks for survival rate prediction in cancer patients and concluded that it is beneficial in cases where expert annotations are not available or difficult to obtain. *One systematic review and meta-analysis:* Guha et al. performed a systematic review and meta-analysis differentiating primary central nervous system lymphoma (PCNSL) from glioblastoma (GBM) using deep learning and radiomics based ML approach. *There are five non-imaging related articles:* Zhu et al. developed transparent machine learning pipeline to efficiently predict Microsatellite instability (MSI), thus, helping pathologists to guide management decisions. Wang et al. conducted a study to reveal the heterogeneity in the tumor microenvironment of pancreatic cancer and analyze the differences in prognosis and immunotherapy responses of distinct immune subtypes. Menon et al. explored the histological similarities across cancers from a deep learning perspective. Huang et al. studied the effects of biofilm nano-composite drugs OMVs-MSN-5-FU on cervical lymph node metastases from oral squamous cell carcinoma (OSCC) on the animal model. Zormpas-Petridis et al. prepared a DL pipeline for mapping tumour heterogeneity on low-resolution whole-slide digital histopathology images. **Figure 1** shows the list of authors based on type of articles submitted towards Research Topic.

## Conclusions

DL has ushered in revolution in the field of oncology research, from cancer screening and diagnosis, to response assessment and survival prediction, thus positively influencing patient management. With the increasing cancer burden and limited number of specialized healthcare providers, there is a growing inclination to use DL at various levels of cancer diagnosis to cater to the needs of patients and the healthcare providers alike. Despite the umpteen benefits, there are a few challenges that DL needs to conquer, before it can be ubiquitously used. Through this Research Topic, we wish to acquaint the readers with the latest ongoing DL based research in cancer diagnosis, which can pave the way for further innovations and research in this field, as full potential of DL is still underutilized.

## Author contributions

All authors listed have made a substantial, direct, and intellectual contribution to the work and approved it for publication.
